# Impact of dental caries on oral health related quality of life among preschool children: perceptions of parents

**DOI:** 10.1186/s12903-021-01396-4

**Published:** 2021-02-15

**Authors:** Mina Pakkhesal, Elham Riyahi, AliAkbar Naghavi Alhosseini, Parisa Amdjadi, Nasser Behnampour

**Affiliations:** 1 Dental Research Center, Community Oral Health Department, School of Dentistry, Golestan University of Medical Sciences, Gorgan, Iran; 2 Dental Research Center, School of Dentistry, Golestan University of Medical Sciences, Gorgan, Iran; 3 Dental Research Center, Orthodontics Department, School of Dentistry, Golestan University of Medical Sciences, Gorgan, Iran; 4 Department of Dental Biomaterials, School of Dentistry, Shahid Beheshti University of Medical Sciences, Tehran, Iran; 5 Department of Biostatistics and Epidemiology, School of Health, Golestan University of Medical Sciences, Gorgan, Iran

**Keywords:** Quality of life, Oral health, Child, Parents

## Abstract

**Background:**

Childhood dental caries can affect the children’s and their parents’ oral health-related quality of life. The aim of the present study was to evaluate the impact of oral and dental health conditions on the oral health-related quality of life in preschool children and their parents.

**Methods:**

In this descriptive-analytical cross-sectional study, samples were selected from children 3 to 6 years old enrolled in licensed kindergartens using "proportional allocation" sampling. Then, the parents of the children were asked to complete the Early Childhood Oral Health Impact Scale (ECOHIS).

**Results:**

In this study, 350 children aged 3 to 6 years were evaluated with a mean age of 4.73 years. The mean dmft index (decayed, missed, and filled teeth) was 3.94 ± 4.17. The mean score of oral health-related quality of life was 11.88 ± 6.9, which 9.36 ± 5.02 belongs to the impact on children and 2.52 ± 3.20 to parents' impact.

**Conclusions:**

The mean score of ECOHIS increased with the dmft index increase in children, indicating a significant relationship between the dmft and ECOHIS score. These outcomes can be used as proper resources to develop preventive policies and promote oral health in young children.

## Background

According to the World Health Organization, the quality of life is defined as “a person’s perceptions of their position in life according to their culture, goals, expectations, standards, and priorities”. Therefore, it is subjective and not observable by others and is based on the person’s understanding of different aspects of life. Therefore, the quality of life of each individual is influenced by their conditional characteristics and social, cultural, and environmental status [1, 2].

Today, health is considered to be a holistic concept that encompasses many aspects, such as physical, emotional, social, and spiritual health. Oral health is also defined as "a comfortable and functional dentition that allows individuals to continue their social role." Therefore, oral health is more than the absence of dental caries or gum disease or even having healthy teeth [3]. Oral health is one of the determinants of quality of life. Overall, the craniofacial complex allows us to talk, laugh, kiss, touch, chew, swallow, and cry. Oral and dental illnesses also cause restrictions in the school, work, and home environments; consequently, hours loss of school and work attendance. Poor oral health can also affect the quality of life. Experiencing pain, tolerating dental abscesses, difficulties in chewing and swallowing, feeling embarrassed about teeth shape or missing teeth, and tooth discoloration or decay may affect daily life and people comfort. In recent years, many studies have been conducted on the impact of oral health on the quality of life [4], especially in young children, because dental caries and traumatic dental injuries (TDI) are the most common problems affecting young children in both developed and developing countries worldwide [5, 6].

Dental caries and dental injuries during childhood may have negatively impact on the oral health-related quality of life of the children and their parents [7–9]. Many of this caries are also left untreated in this age group, which usually affects the weight, growth, quality of life, and cognitive development of the children and may result in hospitalization and emergency dental visits [5]. “Early childhood caries” (ECC) is one such common dental health condition seen in infants and toddlers around the world [10]. Children with untreated early childhood caries (ECC) have significantly poorer oral health related quality of life than children without ECC [11]. Evidence also reveals that ECC results in loss of the workdays for parents to take care of their child or spending time and money in accessing dental care [8]. In addition, parents play an important role in the oral health status of children and in seeking dental care and therefore tend to express feelings of guilt when their child exhibits oral health problems and/or treatment needs [12].

Researchers have used various measurements to assess the Oral Health-Related Quality of Life (OHRQoL), a few of which are designed for children under the age of 6 [5, 13]. These measurements are usually assessed through interviews with children who can speak and write or completed questionnaires by children or their parents. Currently, the Early Childhood Oral Health Impact Scale (ECOHIS) is one of the appropriate tools to assess the oral health related quality of life in children due to their inability to read and write [4, 14].

The questionnaire was initially designed by Pahel [15] and then translated and evaluated for use in other countries like France, China, Brazil, and Iran [15–19].

After translating the Scale into Persian, Dr. Jabbarifar et al. assessed the validity and reliability of the Persian version of the Scale using a questionnaire completed by 246 parents of children aged 2 to 5 years in Tehran and Isfahan. It was concluded that the Persian version of the ECOHIS was valid and reliable to assess the impact of oral health on preschool children’s quality of life of with Persian-speaking parents [20].

Given the importance of patient-centered approaches to clinical decision-making in recent years and the attention paid to the oral health-related quality of life in dentistry, the present study was conducted to investigate the effect of oral health on the quality of life of preschool children and their parents in Gorgan. .

## Methods

The present study was approved by the Research Ethics Committee of Golestan University of Medical Sciences (IR.GOUMS.1397.166) and performed entirely following the Declaration of Helsinki. All participants’ rights were protected. Informed consent was obtained from parents before the study. Moreover, the data were handled anonymously and confidentially in all stages of the study.

### Study population and sampling

The sample size was calculated at 330 preschool children, based on a 0.05 Type I and 0.2 Type II error rate. Also, 20% was added to compensate for possible losses, giving a total sample of 350 preschool children.

This cross-sectional descriptive-analytical study was conducted among 350 children aged 3–6 years selected from about 6000 children registered in the licensed kindergartens of Gorgan. Hence, the list of licensed kindergartens in three municipality districts of Gorgan was prepared. Then, according to the number of children in each municipality district’s kindergartens, the number of children in each district was determined. Numerous kindergartens were selected randomly (allocating a number to each kindergarten and selecting random numbers).

Children aged 3–6 years whose parents could easily speak Persian were selected. Exclusion criteria were a history of systemic diseases or receiving specific medication. Parents who did not complete the questionnaires were also excluded.

In the first session, the aim of research was explained to kindergarten teachers. Then, demographic questionnaires were given to preschool educators and administrators as well as consent forms to be completed by parents. Demographic information included data on the child’s age, gender, ethnicity, birth order, and parental level of education.

### Questionnaires and data collection

In the next session, the parents completed the Persian version of the Early Childhood Oral Health Impact Scale. The questionnaire consists of 13 questions, classified into two sections: “impact on children” and “impact on parents”. The first 9 questions of the questionnaire examine the impact of the children's oral health, including items such as eating, sleeping, and talking. The second section, “impact on parents”, has 4 questions in 2 subscales: parents’ concerns (2 questions) and parents’ functions (2 questions).

Response options included “never”, “hardly ever”, “occasionally”, “often”, “very often”, and “don't know” that received a score of 0 to 5, respectively. A score for the missing items was imputed as an average of the remaining items for each section. Overall, the total score of this index ranges from 0 to 52 with a higher total score indicating more oral health problems and less oral health-related quality of life.

### Children’s oral examination

Clinical examinations were performed by the researcher to measure the dmft index (decayed, missed, and filled teeth) using dental examination tools (disposable dental mirror, dental explorer, sterile gauze, and mask) according to the World Health Organization criteria for the diagnosis of caries. Moreover, all of the oral examinations were performed by a single trained and calibrated researcher. Hence, only intra examiner reliability was determined. Thus the oral examination of 10 randomly selected subjects was repeated at two time points to determine intra examiner reliability. The Kappa coefficient value for intra examiner reliability was 0.87 which is interpreted as very good.

For clinical examination, the child was seated in a chair in front of a window, and a flashlight was used if there was insufficient light. Besides, another person previously trained by the project administrator recorded the codes for the dmft index in the oral health assessment forms (provided by the World Health Organization).

### Data analysis

Data were analyzed by the SPSS software version 16 using mean, standard deviation, frequency, and percentage. Then the normality of the data was determined by the Shapiro–Wilk test. An Independent t-test was used for data with a normal distribution, and the Mann–Whitney test was used for data that did not have a normal distribution. *P*-values less than 0.05 were considered significant.

## Results

In this study, 350 children aged 3–6 years with a mean age of 4.73 years were studied, of whom 189 (54%) were male, and 161 (46%) were female. Also, 228 children were first birth order, 106 were second birth order, and 16 were third or fourth birth order. In terms of ethnicity, 287 children were Fars, 33 were Turkmen, and 30 were Sistani.

The minimum and maximum dmft index of the primary teeth was 0 and 20, respectively with an average of 3.94 and a standard deviation of 4.17. It is noticeable that a higher percentage (89.85%) of the dmft index was related to the decayed teeth (d) component in this study.

According to the results, there was no significant relationship between the mean dmft index and gender and birth order in the family. However, the mean dmft index had a significant relationship with ethnicity, child’s age, and parents’ education level (*P* < 0.05).

In addition, the results showed no significant correlation between the mean score of the oral health-related quality of life and gender, birth order in the family, and ethnicity. Nonetheless, the mean score of the oral health-related quality of life significantly correlated with the child’s age and parents’ education level (*P* < 0.05) (Table 1).

**Table 1:** Mean of dmft score and Impact on oral health-related quality of life according to independent variables

Variables	N (%)	dmft score		*P*-value	Impact on oral health-related quality of life		*P*-value
		Mean	SD		Mean	SD	
*Gender*							
Boys	189 (54%)	3.93	4.22	0.935*	11.27	6/60	0.09**
Girls	161 (46%)	3.96	4.12		12.59	7/22	
*Child’s age*							
2 ≤ age < 3	51 (14%)	1.94	2.28	> 0.0001****	8.21	4.87	> 0.0002***
3 ≤ age < 4	94 (27%)	3.10	3.48		11.54	6.63	
4 ≤ age < 5	104 (30%)	3.84	3.28		12.88	6.68	
5 ≤ age < 6	101 (29%)	6.07	5.23		13	7.67	
*Mother’s education*							
Illiterate/ Elementary	21 (6%)	8.05	5.35	> 0.0001****	13.85	6.62	0.001***
Secondary	18 (5%)	6.38	3.36		16.22	7.28	
Diploma	91 (26%)	4.96	4.39		13.35	7.62	
University	220 (63%)	2.93	3.57		10.72	6.33	
*Father’s education*							
Illiterate/ Elementary	16 (4%)	6.87	4.47	> 0.0001****	15.81	8.19	0.004***
Secondary	34 (10%)	6.35	4.82		13.50	7.45	
Diploma	84 (24%)	5.34	4.68		13.20	7.21	
University	216 (62%)	2.81	3.35		10.81	6.40	
*Ethnicity*							
Fars	287 (82%)	3.60	4.03	0.002****	11.78	6.93	0.783***
Turkmen	33 (9.5%)	4.93	3.73		11.96	7.06	
Sistani	30 (8.5%)	6.16	5.16		12.66	6.81	
*Birth order*							
First child	228 (65%)	3.83	3.98	0.457***	11.81	6.82	0.450***
Second child	106 (30%)	3.97	4.54		12.26	7.23	
Third child	16 (5%)	5.43	4.21		10.31	6.20	

^*^Calculated by independent T-test

^**^Calculated by Mann–Whitney test

^***^Calculated by Kruskal Wallis test

^****^Calculated by analysis of variance (ANOVA) test

The mean score of the oral health-related quality of life was 11.88 ± 6.91 (range 0–33); it was 9.36 ± 5.02 (range 0–25) in the child impact and 2.52 ± 3.20 (range 0–16) in the parents’ impact (Table 2).

**Table 2:** ECOHIS responses in the survey of parents of 3–6 year-olds (N = 350)

Impacts	Never N (%)	Hardly ever N (%)	Occasionally N (%)	Often N (%)	Very often N (%)	Don’t know N (%)
*Child impacts*						
Oral/ dental pain	169 (48.3)	75 (4.21)	58 (6.16)	35 (10)	12 (4.3)	1 (3.0)
Difficulty drinking	249 (1.71)	48 (7.13)	23 (6.6)	16 (6.4)	8 (3.2)	6 (7.1)
Difficulty eating	204 (3.58)	66 (9.18)	36 (3.10)	22 (3.6)	16 (6.4)	6 (7.1)
Difficulty pronouncing words	186 (1.53)	95 (1.27)	21 (6)	22 (3.6)	19 (4.5)	7 (2)
Missed preschool or school	32 (1.9)	87 (9.24)	109 (1.31)	74 (1.21)	43 (3.12)	5 (4.1)
Trouble sleeping	61 (4.17)	124 (4.35)	67 (1.19)	52 (9.14)	41 (7.11)	5 (4.1)
Irritable or frustrated	94 (9.26)	117 (4.33)	73 (9.20)	41 (7.11)	21 (6)	4 (1.1)
Avoided smiling or laughing	154 (44)	101 (9.28)	27 (7.7)	15 (3.4)	10 (9.2)	43 (3.12)
Avoided talking	180 (51.4)	82 (23.4)	27 (7.7)	16 (4.6)	10 (2.9)	35 (10)
*Parents impact*						
Been upset	186 (1.53)	82 (4.23)	42 (12)	27 (7.7)	8 (3.2)	5 (4.1)
Felt guilty	218 (3.62)	78 (3.22)	17 (9.4)	16 (6.4)	12 (4.3)	9 (6.2)
Time off from work and home	240 (6.68)	45 (9.12)	41 (7.11)	12 (4.3)	8 (3.2)	4 (1.1)
Financial impact	225 (3.64)	50 (3.14)	35 (10)	16 (5.4)	8 (3.2)	16 (6.4)

**Fig. 1:**
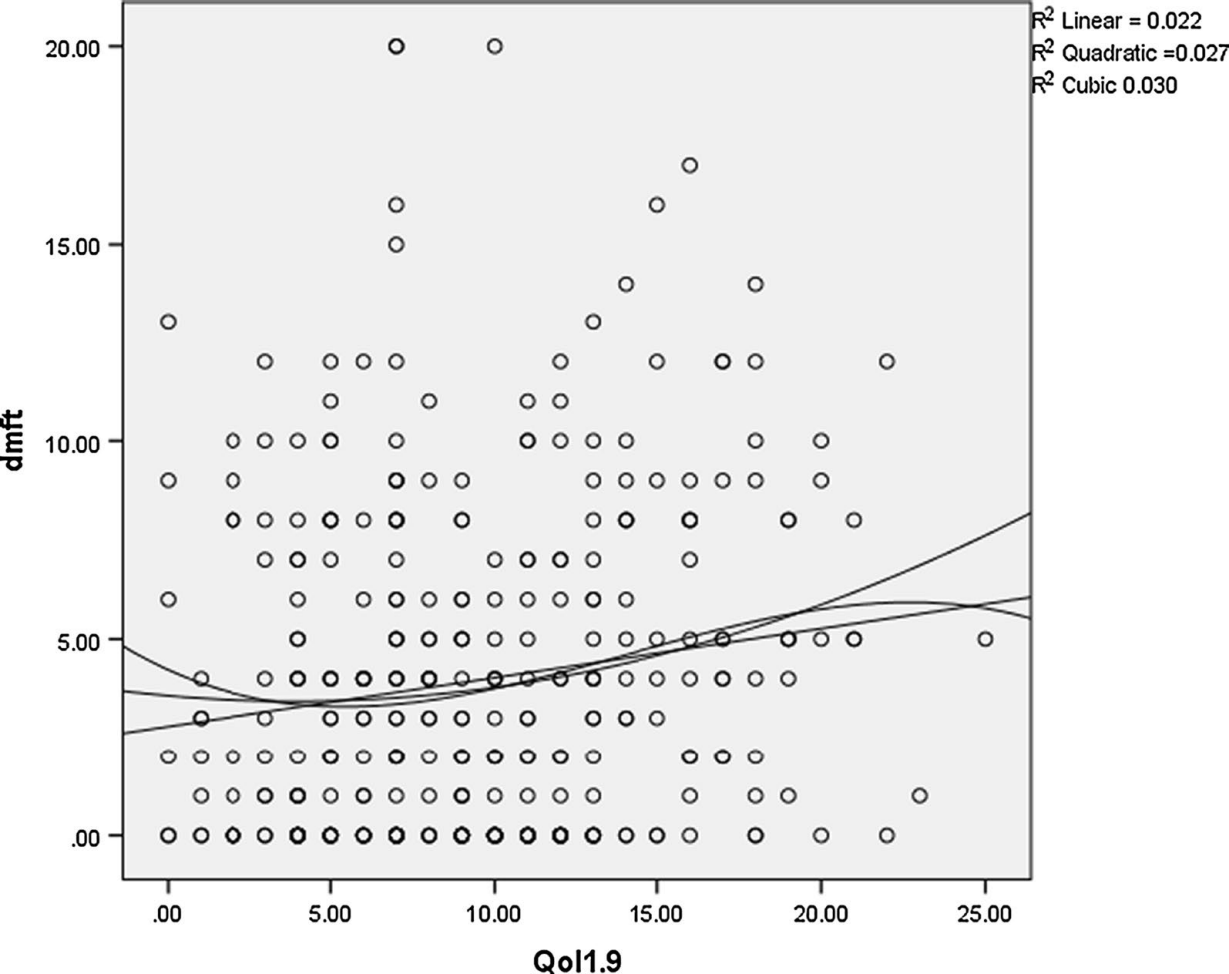
Scatter plot of relation between dmft score and ECOHS in child impact

**Fig. 2:**
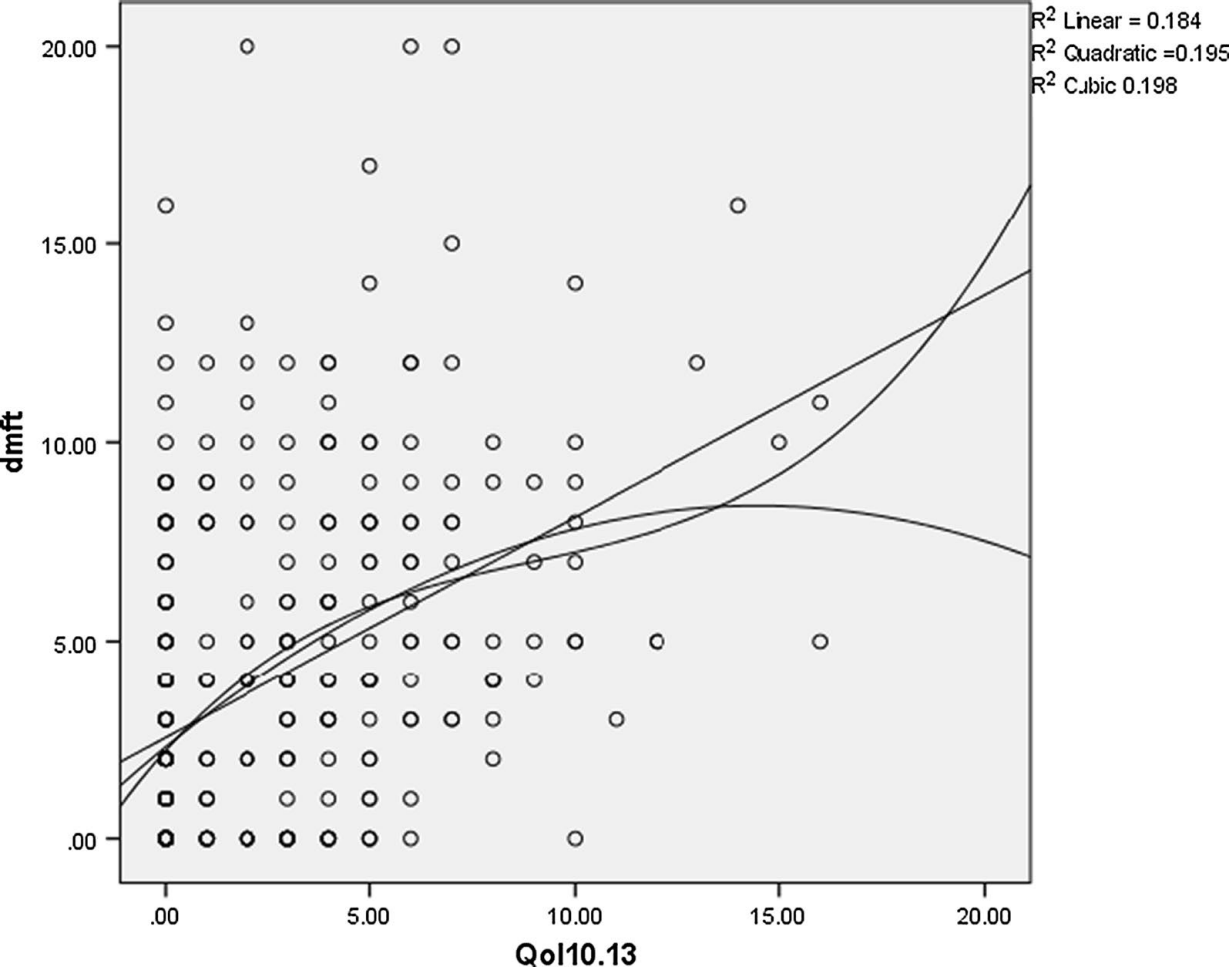
Scatter plot of relation between dmft score and ECOHS in parents impact

According to the results, with an increase in the children’s dmft index, the mean score of oral health-related quality of life increased, too (Table 3). This relation was more robust in the family impact compared to the child impact (Figs. 1, 2). It should be noted that an increase in the mean score of quality of life indicated a poorer oral health status.

**Table 3:** Impact of severity of caries on oral health-related quality of life—child and family impact section

	dmft = 0 (Caries free)	1 ≤ dmft ≤ 5	dmft ≥ 6	Total	*P*-value
N (%)	111 (31.7%)	136 (38.9%)	103 (29.4%)		
ECOHIS (Child impact section)	8.42 ± 4.24	9.66 ± 5.30	9.96 ± 5.32	9.36 ± 5.02	0.05*
ECOHIS (Family impact section)	0.87 ± 1.70	2.72 ± 3.25	4.01 ± 3.57	2.52 ± 3.20	0.000*
Total	9.29 ± 5.04	12.38 ± 8.25	13.97 ± 8.89	11.88 ± 6.91	

^*^ Calculated by Analysis of variance (ANOVA) test

## Discussion

In the present study, the mean score of oral health-related quality of life was 11.88 ± 6.91 in preschool children of Gorgan, which is in agreement with a study by Amirabadi et al. [21] in preschool children of Zahedan (10.94 ± 7.69) and another study in preschool children of Babol (6.65 ± 3.57) [22]. Moreover, the results of a study by Sajjadi et al. in Kerman preschool children [23] showed that the ECOHIS score was 4.07 ± 0.79 for children and 3.28 ± 0.83 for their parents. Shaghagheian et al. study [24] reported an ECOHIS score of 19.46 ± 8.42 for preschool children in Shiraz. Discrepancies between our results and previous study can be explained by the use of different scores of the responses and analysis for ECOHIS score. In the present study, the scores of the responses according to the original questionnaire (ECOHIS) [15] ranged from 0 to 4 and thus, the total scores ranged from 0 to 52, while in some of the above studies, the scores of the responses ranged from 1 to 5 and therefore the sum of the scores ranged from 13 to 65. Given that a lower score indicates a better oral health-related quality of life, it seems that the participants had a relatively good quality of life.

The mean dmft index was 3.93 ± 4.22 in the present study, 1.54 ± 2.47 in a study by Segovia-Villanueva et al. in Mexico [25] and 2.1 ± 3.1 in a study by Scarpelli et al. in Brazil [19]. Based on these findings, the children that participated in the present study had poorer dental health than the studies mentioned above. However, the recent study participants had better scores compared to the mean dmft index of Babol (4.39 ± 3.69) and Kerman (5.6 ± 3.6) preschool children [22, 23].

The present study results showed that with an increase in the mean dmft index, the total score of oral health-related quality of life increased as well. Our results are also in accordance with previous studies assessing the impact of dental caries on preschool children’s OHRQoL [7–10]. This effect was greater on the parents’ quality of life than that of children, indicating that the impact of children's oral health on parents' quality of life was greater than its effect on their quality of life. The reason for this finding is the importance of the children’s health for parents. The parents are usually very sensitive to their children's health while a child might lack the perspective and insight; thus, the parents’ quality of life would be more affected than that of children. Also, Sakaryali et al. study showed that both simple and severe conditions of ECC cause aesthetic and functional problems in children, and also deal the daily life of parents [8].

Moreover, in accordance with Paula et al. [26] study results the mean score of quality of life related to children's oral health decreased with increased parents’ education level. In Kumar et al. study, children of high-income and high-educated families had the better oral health-related quality of life [27]. Moreover, Nanayakkara found that children whose fathers were less educated had higher dmft scores, and a worse oral health-related quality of life [28]. Also, our results were in line with a Diaz et al. [7] study that found mother’s education association with better preschool children’s OHRQoL according to the Colombian ECOHIS. Considering the greater impact of the mother’s education than father’s education, which was also evident in the present study, it can be concluded that mothers play more effective role in improving the oral health-related quality of life in children. Sajjadi et al. [23] found that the OHRQoL only increased with an increase in mother’s education while father’s education had no significant relationship with the OHRQoL. It is usually expected that increased general and specialized knowledge may lead to increased health awareness, including oral health, or make parents more concerned about their children's oral health. On the other hand, lower education levels can lead to a lower income, unemployment, and poor working conditions, which may affect health-related behaviors and oral health status.

Also, similar to present study results, Nemati et al. [22] found no significant difference in the effect of oral health on quality of life between boys and girls. The reason is that the children evaluated in this study were very young (preschool), and gender differences in these children may have not yet influenced their understanding of the aesthetic aspects of oral health.

The results of this study showed a significant relationship between the mean score of oral health-related quality of life and age, which was consistent with the results of a study by Li et al. in which the quality of life had a significant relationship with age, i.e. an increase in age increased the impact of oral health on the quality of life [29]. Decreased oral health-related quality of life at higher ages is not unexpected because the teeth are more likely to become exposed to risk factors with age, and therefore the children will suffer more. In other words, problems such as chewing and speaking are more prominent at 5–6 years of age than 3 to 4 years; on the other hand, the persistence of the problem until higher ages will draw the parents’ attention to it. Another finding of our study in line with the sakaryali et al. [8] study showed that the child’s birth order in the family was not significantly correlated with the OHRQoL.

This study suffered from limitations; for example, some children did not cooperate for their dental examinations, some parents were unwilling to answer some questions, and some kindergarten authorities were not cooperative.

Moreover, the current study was accomplished only on kindergartens' preschool children. A further population-based research would be required to OHRQoL assessment of preschool children living in the Gorgan city to confirm the present study results.

## Conclusion

The present study showed that the oral health status of Gorgan preschool children affected their own and their parents’ quality of life. The mean score of ECOHIS increased with the dmft index increase in children, indicating a significant relationship between the dmft and ECOHIS score.

These results can be used as proper resources to develop preventive policies and promote oral health in young children at a national level.

## Abbreviations

dmft: decayed, missed, filled teeth
ECOHIS: Early Childhood Oral Health Impact Scale
OHRQoL: Oral health-related quality of life
WHO: World Health Organization

## Availability of data and material

The datasets used and/or analyzed during the current study are available from the corresponding author on reasonable request.

## References

[1] Bonomi AE, Patrick DL, Bushnell DM, Martin M (2000). Validation of the United States' version of the World Health Organization Quality of Life (WHOQOL) instrument. J Clin Epidemiol.

[2] Organization WH (1996). WHOQOL-BREF: introduction, administration, scoring and generic version of the assessment: field trial version, December 1996.

[3] Daly B, Batchelor P, Treasure E, Watt R (2013). Essential dental public health.

[4] Petersen PE (2003). The World Oral Health Report 2003: continuous improvement of oral health in the 21st century—the approach of the WHO Global Oral Health Programme. Commun Dent Oral Epidemiol.

[5] Abanto J, Tsakos G, Paiva SM, Carvalho TS, Raggio DP, Bönecker M (2014). Impact of dental caries and trauma on quality of life among 5-to 6-year-old children: perceptions of parents and children. Commun Dent Oral Epidemiol.

[6] Gomes MC, de Almeida Pinto-Sarmento TC, de Brito Costa EMM, Martins CC, Granville-Garcia AF, Paiva SM (2014). Impact of oral health conditions on the quality of life of preschool children and their families: a cross-sectional study. Health Qual Life Outcomes.

[7] Díaz S, Mondol M, Peñate A, Puerta G, Boneckér M, Martins Paiva S (2018). Parental perceptions of impact of oral disorders on Colombian preschoolers' oral health-related quality of life. Acta Odontol Latinoam: AOL.

[8] Sakaryali D, Bani M, Cinar C, Alacam A (2019). Evaluation of the impact of early childhood caries, traumatic dental injury, and malocclusion on oral health-related quality of life for Turkish preschool children and families. Niger J Clin Pract.

[9] Rajab LD, Abdullah RB (2020). Impact of dental caries on the quality of life of preschool children and families in Amman, Jordan. Oral Health Prev Dent.

[10] Mansoori S, Mehta A, Ansari MI (2019). Factors associated with oral health related quality of life of children with severe—Early Childhood Caries. J Oral Biol Craniofac Res.

[11] Sheiham A (2006). Dental caries affects body weight, growth and quality of life in pre-school children. Br Dent J.

[12] Gomes MC, Clementino MA, Pinto-Sarmento TC, Martins CC, Granville-Garcia AF, Paiva SM (2014). Association between parental guilt and oral health problems in preschool children: a hierarchical approach. BMC Public Health.

[13] McGrath C, Broder H, Wilson-Genderson M (2004). Assessing the impact of oral health on the life quality of children: implications for research and practice. Commun Dent Oral Epidemiol.

[14] Barbosa TDS, Gavião MBD (2009). Evaluation of the family impact scale for use in Brazil. J Appl Oral Sci..

[15] Pahel BT, Rozier RG, Slade GD (2007). Parental perceptions of children's oral health: the Early Childhood Oral Health Impact Scale (ECOHIS). Health Qual Life Outcomes.

[16] Tesch FC, Oliveira BHD, Leão A (2008). Semantic equivalence of the Brazilian version of the early childhood oral health impact scale. Cadernos de saude publica.

[17] Lee GH, McGrath C, Yiu CK, King NM (2009). Translation and validation of a Chinese language version of the Early Childhood Oral Health Impact Scale (ECOHIS). Int J Pediatr Dent.

[18] Lee GH, McGrath C, Yiu CK, King NM (2011). Sensitivity and responsiveness of the Chinese ECOHIS to dental treatment under general anaesthesia. Commun Dent Oral Epidemiol.

[19] Scarpelli AC, Oliveira BH, Tesch FC, Leão AT, Pordeus IA, Paiva SM (2011). Psychometric properties of the Brazilian version of the early childhood oral health impact scale (B-ECOHIS). BMC Oral Health.

[20] Jabarifar S-E, Golkari A, IJadi MH, Jafarzadeh M, Khadem P (2010). Validation of a Farsi version of the early childhood oral health impact scale (F-ECOHIS). BMC Oral Health.

[21] Amirabadi F, Rahimian-Imam S, Ramazani N, Saravani S, Kameli S. Evaluation of dental status and its association with oral health-related quality of life in preschool children in Zahedan City, Iran: a cross-sectional study. Middle East J Rehabil Health. 2017;4(1):1–5. e37043. 10.17795/mejrh-37043.

[22] Nemati S, Ghasempour M, Khafri S (2016). Impact of oral and dental health on quality of life in Iranian preschool children and their families. Electron Phys.

[23] Sajadi FS, Pishbin L, Azhari SH, Moosazadeh M (2015). Impact of oral and dental health on children’s and parents’ quality of life based on Early Childhood Oral Health Impact Scale (ECOHIS) Index. Int J Dent Sci Res.

[24] Shaghaghian S, Bahmani M, Amin M (2015). Impact of oral hygiene on oral health-related quality of life of preschool children. Int J Dent Hyg.

[25] Segovia-Villanueva A, Estrella-Rodríguez R, Medina-Solís CE, Maupomé G (2006). Dental caries experience and factors among preschoolers in Southeastern Mexico: a brief communication. J Public Health Dent.

[26] Paula JS, Leite IC, Almeida AB, Ambrosano GM, Pereira AC, Mialhe FL (2012). The influence of oral health conditions, socioeconomic status and home environment factors on schoolchildren's self-perception of quality of life. Health Qual Life outcomes.

[27] Kumar S, Kroon J, Lalloo R (2014). A systematic review of the impact of parental socio-economic status and home environment characteristics on children’s oral health related quality of life. Health Qual Life Outcomes.

[28] Nanayakkara V, Renzaho A, Oldenburg B, Ekanayake L (2013). Ethnic and socio-economic disparities in oral health outcomes and quality of life among Sri Lankan preschoolers: a cross-sectional study. Int J Equity Health.

[29] Li S, Veronneau J, Allison PJ (2008). Validation of a French language version of the early childhood oral health impact scale (ECOHIS). Health Qual Life Outcomes.

